# Synthetic hematocrit derived from the longitudinal relaxation of blood can lead to clinically significant errors in measurement of extracellular volume fraction in pediatric and young adult patients

**DOI:** 10.1186/s12968-017-0377-z

**Published:** 2017-08-02

**Authors:** Frank J. Raucci, David A. Parra, Jason T. Christensen, Lazaro E. Hernandez, Larry W. Markham, Meng Xu, James C. Slaughter, Jonathan H. Soslow

**Affiliations:** 1 Department of Pediatrics, Thomas P. Graham Jr, Division of Pediatric Cardiology, Vanderbilt University Medical Center, Nashville, USA; 2 Joe DiMaggio Children’s Hospital, Pediatric and Congenital Cardiac MRI program, Los Angeles, CA USA; 3 Department of Biostatistics, Vanderbilt University Medical Center, Nashville, TN USA; 4 Thomas P. Graham, Jr. Division of Pediatric Cardiology, Monroe Carell Jr. Children’s Hospital at Vanderbilt, 2200 Children’s Way, Suite 5230, Doctors’ Office Tower, Nashville, TN 37232-9119 USA

**Keywords:** Extracellular volume fraction, T1 mapping, Cardiovascular magnetic resonance, Cardiomyopathy, Modified look-locker inversion (MOLLI)

## Abstract

**Background:**

Extracellular volume fraction (ECV) is altered in pathological cardiac remodeling and predicts death and arrhythmia. ECV can be quantified using cardiovascular magnetic resonance (CMR) T1 mapping but calculation requires a measured hematocrit (Hct). The longitudinal relaxation of blood has been used in adults to generate a synthetic Hct (estimate of true Hct) but has not been validated in pediatric populations.

**Methods:**

One hundred fourteen children and young adults underwent a total of 163 CMRs with T1 mapping. The majority of subjects had a measured Hct the same day (*N* = 146). Native and post-contrast T1 were determined in blood pool, septum, and free wall of mid-LV, avoiding areas of late gadolinium enhancement. Synthetic Hct and ECV were calculated and intraclass correlation coefficient (ICC) and linear regression were used to compare measured and synthetic values.

**Results:**

The mean age was 16.4 ± 6.4 years and mean left ventricular ejection fraction was 59% ± 9%. The mean measured Hct was 41.8 ± 3.0% compared to the mean synthetic Hct of 43.2% ± 2.9% (*p* < 0.001, ICC 0.46 [0.27, 0.52]) with the previously published model and 41.8% ± 1.4% (*p* < 0.001, ICC 0.28 [0.13, 0. 42]) with the locally-derived model. Mean measured mid-free wall ECV was 30.5% ± 4.8% and mean synthetic mid-free wall ECV of local model was 29.7% ± 4.6% (*p* < 0.001, ICC 0.93 [0.91, 0.95]). Correlations were not affected by heart rate and did not significantly differ in subpopulation analysis. While the ICC was strong, differences between measured and synthetic ECV ranged from −8.4% to 4.3% in the septum and −12.6% to 15.8% in the free wall. Using our laboratory’s normal cut-off of 28.5%, 59 patients (37%) were miscategorized (53 false negatives, 6 false positives) with published model ECV. The local model had 37 miscategorizations (20 false negatives, 17 false positives), significantly fewer but still a substantial number (23%).

**Conclusions:**

Our data suggest that use of synthetic Hct for the calculation of ECV results in miscategorization of individual patients. This difference may be less significant once synthetic ECV is calculated and averaged over a large research cohort, making it potentially useful as a research tool. However, we recommend formal measurement of Hct in children and young adults for clinical CMRs.

**Electronic supplementary material:**

The online version of this article (doi:10.1186/s12968-017-0377-z) contains supplementary material, which is available to authorized users.

## Background

Extracellular matrix (ECM) expansion from edema and fibrosis is a pathological finding in many forms of cardiovascular disease. Cardiovascular magnetic resonance (CMR) mapping of the longitudinal relaxation time constant (T1) can be used to assess these myocardial abnormalities. Extracellular volume fraction (ECV) is a quantitative analysis derived from the pre- and post-contrast T1 maps of the myocardium and blood pool that correlates strongly with histological measures of ECM expansion [1–4]. This technique has recently been gaining favor as a biomarker in multiple myocardial disease processes [5–11]. Additionally, ECV has been shown to correlate with clinical outcomes, including arrhythmia, heart failure, and mortality [6, 7, 9–17].

The hematocrit (Hct) is related to the blood volume of distribution and converts the equation from a partition coefficient calculation to a myocardial ECV using the term (100 – Hct) in the following equation (∆ represents the difference between post- and pre-contrast T1):

ECV $$ =\left(100-Hct\right)\frac{\Delta \left(\frac{1}{\begin{array}{c}\mathrm{T}1 myocardium\\ {}\ \end{array}}\right)}{\Delta \left(\frac{1}{\begin{array}{c}\mathrm{T}1 blood\\ {}\ \end{array}}\right)} $$

Calculating ECV requires measurement of Hct, which can be burdensome, especially in pediatric populations. Cost may also be a limiting factor for CMRs performed for research purposes as separate funding must be available and can be particularly expensive for large studies. Finally, as hematocrit is not measured routinely in the setting of outpatient CMRs, retrospective analyses of ECV are difficult to perform. Recently, Treibel and colleagues have used the linear relationship between Hct and the native T1 of blood pool to derive a linear regression model to estimate synthetic Hct and ECV [18]. This model showed strong correlation in a separate, large outcome cohort of adults; however this has not been evaluated in the literature in pediatric populations. Additionally, synthetic Hct and ECV calculations are now being incorporated in-line in CMR software packages. The goal of the present study is to determine if use of synthetic Hct can be used to reliably estimate ECV in clinical settings for pediatric populations.

## Methods

### Patient selection

This single-center, retrospective, observational study was approved by the Vanderbilt University Institutional Review Board and completed between January 2013 and February 2017. Consent was obtained for all participants. Patients who underwent a CMR using T1 mapping with modified Look-Locker inversion (MOLLI) recovery sequence and who had a Hct drawn within 3 months of the date of CMR were eligible and all Hct values were determined in a central laboratory. Pediatric, young adult, and adult congenital patients were included. None of the patients in this study had general anesthesia and none of the patients received intravenous fluids aside from the small volume administered as part of the contrast injection. Pertinent clinical data, including underlying cardiac diagnosis, were collected.

### CMR protocol

CMR was performed on a 1.5 Tesla Siemens Avanto (Siemens Healthcare, Erlangen, Germany) with an 8 channel cardiac coil. Functional imaging was performed as previously described using balanced steady state free precession (bSSFP) imaging [19]. Breath-held MOLLI sequences were performed prior to and 15 min after contrast administration at the level of the base, mid-LV, and apex in the short axis plane [20, 21]. MOLLI sequences were motion-corrected, ECG-triggered images obtained in diastole with typical imaging parameters: non-selective inversion with a 35° flip angle, single shot SSFP imaging, initial inversion time of 120 ms with 80 ms increments, field of view 340 × 272 mm^2^, matrix size 256 × 144, slice thickness 8 mm, voxel size 1.3 × 1.9 × 8.0 mm^3^, TR/TE 2.6 ms/1.1 ms, parallel imaging factor of two. The matrix size was decreased to 192 × 128 for heart rates >90 (approximate voxel size 1.8 × 2.1 × 8 mm^3^). Intravenous Gd-DTPA contrast (gadopentate dimeglumine or gadobutrol, Bayer Healthcare Pharmaceuticals, Wayne, NJ, USA) was administered through a peripheral intravenous line (PIV) at a dose of 0.2 mmol/kg. The pre-contrast MOLLI acquired 5 images after the first inversion with a 3 beat pause followed by 3 images after the second inversion, or 5(3)3 [22]. Early in the protocol, this was modified to the equivalent of a 3 s pause, or 5(3 s)3, to reduce bias from higher heart rates (3 beat pause for heart rate of 60, 4 beat pause for heart rate of 80, and so on). The post-contrast protocol was acquired at a 4(1)3(1)2. Motion correction as described by Xue, et al. was performed and a T1 map was generated on the scanner [23]. One of the investigators was present for all scans and reviewed the MOLLI sequences with the technologist for adequacy at the time of the scan. Any image felt to be inadequate due to poor breath holds or poor motion correction was repeated at the time of the scan.

### T1 analysis and ECV quantification

One reader (FR) traced all contours in this study. Regions of interest (ROIs) were manually drawn on native and post-contrast T1 images in blood pool, septum, and free wall of mid-LV. ROIs of the myocardium were carefully contoured so as to only include the mid-myocardium, avoiding contamination with blood pool or epicardial fat. Given the significant variability in underlying cardiovascular disease that could include patients with myocardial ischemia, areas of LGE were not included in the ROIs. Synthetic Hct was calculated using the published model [18]: [Hct_MOLLI_ = (866.0 · [1/T1_blood_]) - 0.1232]. A locally-derived linear regression model was also created (see Results for details). ECVs were calculated as described above at the mid-septum and mid-LV free wall using both measured and synthetic Hct values. Finally, to evaluate whether the synthetic Hct provides added value over simply assigning the same Hct to all patients (equivalent to the partition coefficient), the ECV was recalculated in all patients after assigning Hct values of both 40% and 45% (reported as static Hct). The Hct of 45% provided ECV values closest to the measured ECV, so those results are reported below.

ECV results were compared to our laboratories normal cut-off of 28.5%. This cut-off was conservatively set at 3 standard deviations above the mean ECV derived from a previously published cohort of healthy young adult patients [8]. For comparison, analysis was repeated for a normal cut-off of 27.0%, representing 2 standard deviations above the mean ECV.

### Statistical analysis

Continuous data are presented as a mean and standard deviation. A locally derived synthetic ECV was created from the longitudinal relaxivity of blood (R1, or 1/T1). This model was created using linear regression modeling with R1 as the predictor variable and the measured hematocrit as the outcome. All three models (published, local, and static) were compared with measured values using multiple statistical methods. Correlations between synthetic and measured Hct and synthetic and measured ECVs were evaluated using linear regression modeling. Agreement between individual sets of synthetic and measured variables were represented graphically using Bland-Altman plots [24]. This allowed for assessment of intrinsic bias in each model. Intraclass correlation coefficients were used to determine how strongly synthetic and measured Hct and ECVs correlated overall and within subgroups. It was assumed that repeated CMRs from the same subject were independent. Analyses were performed with SPSS (v 24, International Business Machines, Inc. Chicago, Illinois, USA). Study data were collected and managed using REDCap (Research Electronic Data Capture) electronic data capture tools hosted at Vanderbilt [25].

## Results

### Demographics

A total of 163 CMRs from 114 children and young adults were included in the study. The characteristics for each participant at each CMR are shown in Table 1. Demographics are for individual patient characteristics at each CMR. The average age at the time of CMR was 16.4 ± 6.4 years. All but 17 patients had Hct drawn on the same day as the CMR. There were a disproportionate number of patients with muscular dystrophy in our cohort as this is the primary population for which our center reports ECV. This also led to a male predominance in our study population.

**Table 1:** Patient characteristics

	*n* or mean ± SD	Portion or Range
Age (years)	16.4 ± 6.4	(7.4, 47.7)
Gender		
Male	145	(89%)
Female	18	(11%)
BSA (m^2^)	1.61 ± 0.39	(0.77, 2.62)
Heart Rate (bpm)	93 ± 21	(45, 150)
LVEF (%)	59 ± 9	(24, 84)
LV EDVI (ml/m^2^)	70.6 ± 26.9	(29.4208.6)
LV ESVI (ml/m^2^)	29.6 ± 18.4	(7.9, 153.7)
Same Day Hct		
Yes	146	(90%)
No	17	(10%)
Primary Diagnosis		
Congenital		
Arch abnormalities	5	(3%)
Aortic valve anomalies	7	(4%)
ASD	1	(0.6%)
AVSD	2	(1.2%)
Mitral valve anomalies	1	(0.6%)
Pulmonary valve anomalies	5	(3%)
ToF	4	(2.5%)
Tricuspid anomalies	1	(0.6%)
VSD	2	(1.2%)
DILV	1	(0.6%)
DORV	1	(0.6%)
CCTGA	1	(0.6%)
D-TGA	1	(0.6%)
Other	2	(1.2%)
Total	24	(15%)
Secondary/Acquired		
Arrhythmia	2	(1.2%)
Cardiomyopathy	11	(7%)
Chemotherapy	6	(4%)
Kawasaki Disease	1	(0.6%)
Muscular Dystrophy	86	(53%)
Myocarditis	9	(5%)
Other acquired	4	(3%)
Total	119	(73%)
Normal/Control	18	(11%)

BSA = Body surface area; LVEF = left ventricular ejection fraction; LV EDVI = left ventricular end-diastolic volume indexed; LV EDSI = left ventricular end-systolic volume indexed; ASD = atrial septal defect; AVSD = atrioventricular septal defect; ToF = tetralogy of Fallot; VSD = ventricular septal defect; DILV = double inlet left ventricle; DORV = double outlet right ventricle; CCTGA = congenitally corrected transposition of the great arteries; D-TGA = D-transposition of the great arteries

### Published model demonstrates strong ECV correlation

**Fig. 1:**
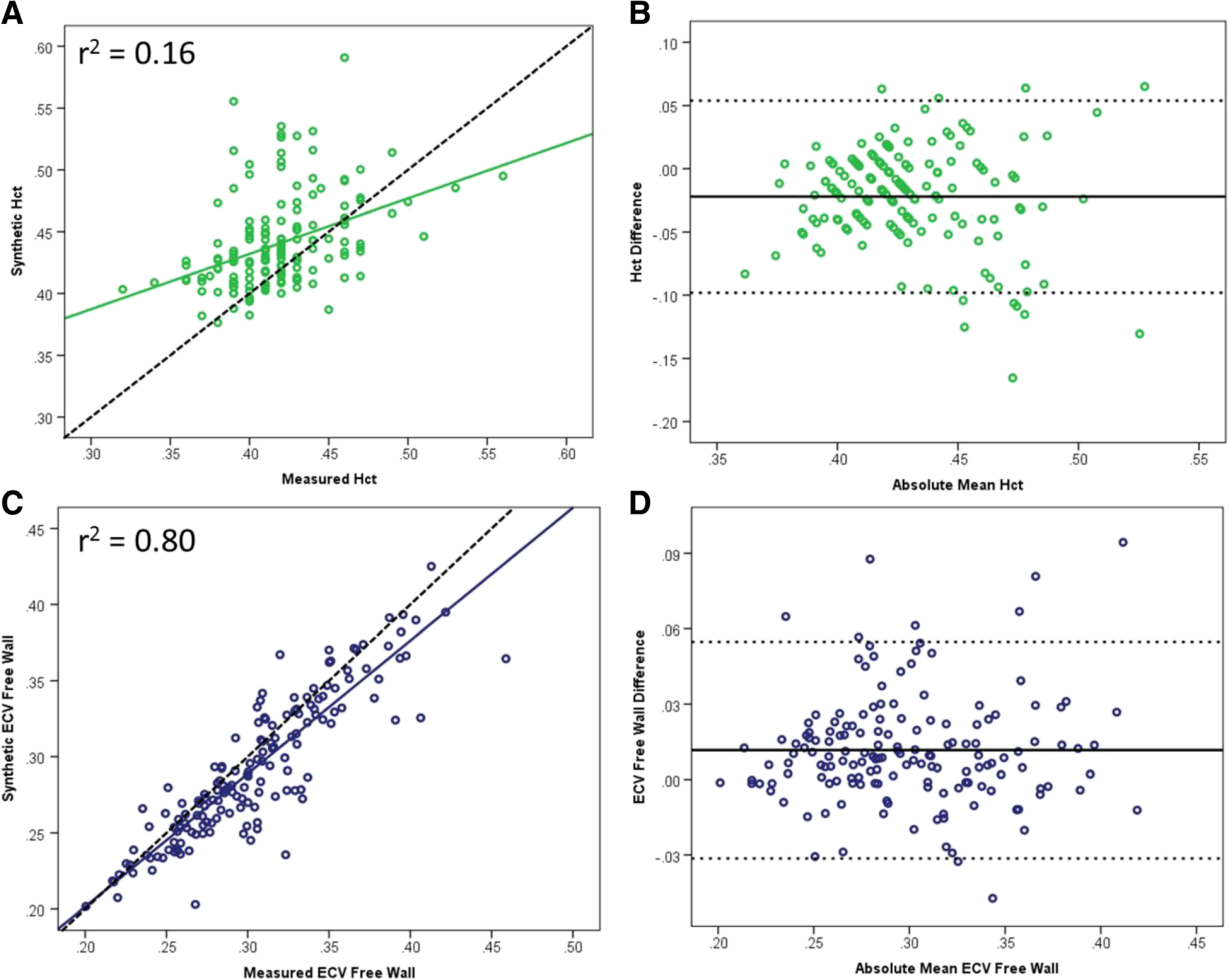
Linear regression fit of measured vs synthetic Hct and ECV for published model. Poor regression fit to the published model (**a**) with a bias of 2.2% on Bland-Altman analysis (**b**). However at the mid-free wall, the regression fit for ECV is substantially better (**c**) with an expected inverse bias on Bland-Altman analysis due to the bias seen in the Hct (**d**). Dashed line in A and C represents line of identity. For Bland-Altman plots, solid line represents mean difference and dashed lines (B and D) are ±1.96SD

Using the equation for calculation of synthetic Hct published by Treibel and colleagues [18] for T1 MOLLI sequences, a mean synthetic Hct of 44.0% ± 3.7% was calculated (Table 2). The mean measured Hct for the cohort was 41.8% ± 3.4% and there was poor correlation between synthetic and measured Hcts by linear regression modeling (r^2^ = 0.16, *p* < 0.001, Fig. 1a). Bland-Altman analysis demonstrated a − 2.2% bias (Fig. 1b) in the calculated synthetic Hct. The difference between measured and synthetic Hct ranged from −16.6% to 6.5%. Despite this poor correlation for the Hct, there was a strong correlation between measured and synthetic ECVs at the mid-free wall of the left ventricle (r^2^ = 0.80, *p* < 0.001, Fig. 1c) and the mid-septum (r^2^ = 0.72, *p* < 0.001, Additional file 1: Figure S1A). Bland-Altman analysis demonstrated a small but significant bias in the synthetic mid-free wall ECV (Figs. 1d) and mid-septum ECV of 1.2% (Additional file 1: Figure S1B). This bias was expected given the bias in the synthetic Hct and its relationship with ECV. There was significant variation in the difference between measured and synthetic ECV, ranging from −4.5% to 10.2% for the mid-septum and from −4.7% to 9.4% for the mid-free wall.

**Table 2:** Comparison of measured to synthetic Hct and ECV for each regression fit

	Mean ± SD	Range
Measured Hct (%)	41.8 ± 3.4	(32, 56)
Synthetic Hct_published_ (%)	44.0 ± 3.7	(37.6, 59.1)
Synthetic Hct_local_ (%)	41.8 ± 1.4	(39.5, 57.3)
Measured ECV Mid-septum (%)	29.5 ± 3.9	(20.8, 46.2)
Synthetic ECV_published_ Mid-septum (%)	28.4 ± 3.7	(20.6, 45.7)
Synthetic ECV_local_ Mid-septum (%)	29.5 ± 3.6	(21.2, 46.2)
Synthetic ECV_static_ Mid-septum (%)	27.9 ± 3.5	(19.9, 43.1)
Measured ECV Mid-free wall (%)	30.5 ± 4.8	(20.1, 45.9)
Synthetic ECV_published_ Mid-free wall (%)	29.3 ± 4.7	(20.2, 42.5)
Synthetic ECV_local_ Mid-free wall (%)	29.7 ± 4.6	(19.6, 41.7)
Synthetic ECV_static_ Mid-free wall (%)	28.8 ± 4.4	(17.8, 41.4)

### Local regression model demonstrates excellent ECV correlation

**Fig. 2:**
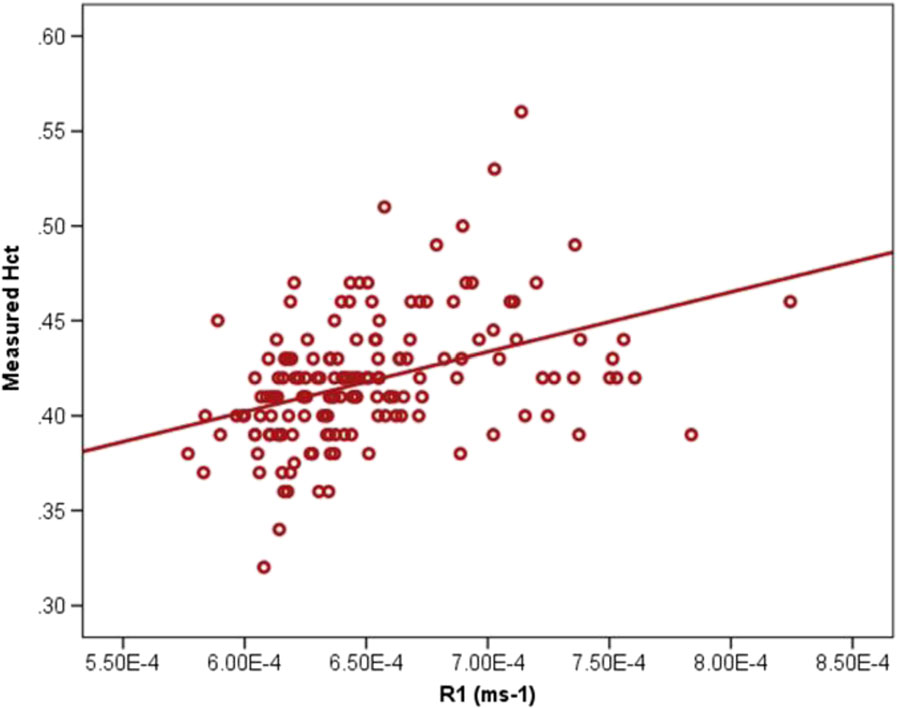
Linear Regression for native T1 blood pool vs measured Hct. The local model was derived from linear regression of the blood pool native T1 (R1) and measured Hct

**Fig. 3:**
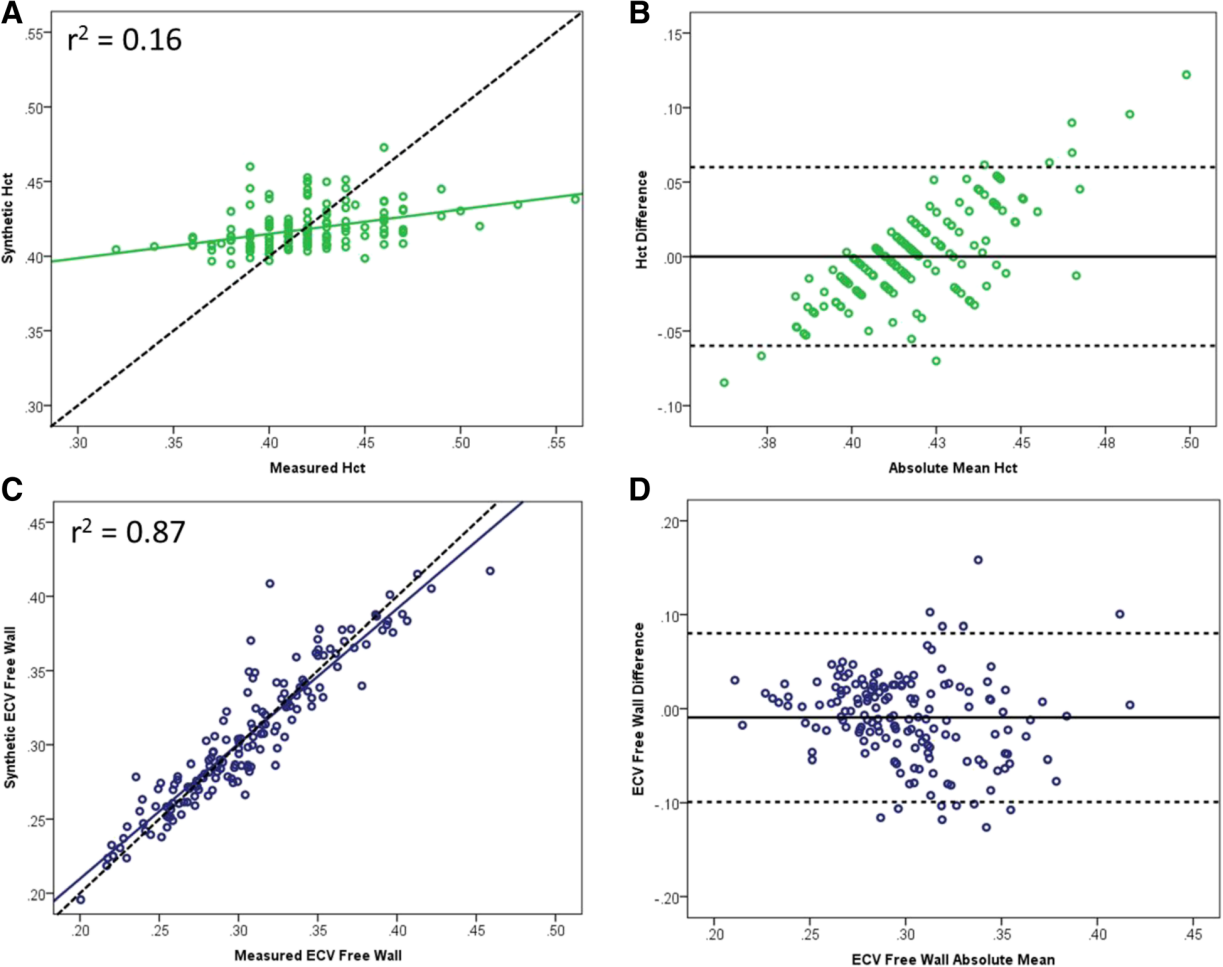
Linear regression fit of measured vs synthetic Hct and ECV for local model. Similarly poor regression fit using the local model (**a**) with elimination of bias on Bland-Altman analysis (**b**). There was a tendency for the local equation to overestimate Hct at higher values and underestimate Hct at lower values. However there was excellent fit for measured vs synthetic ECV at the mid-free wall (**c**) without significant bias on Bland-Altman analysis (**d**). Dashed line in A and C represents line of identity. For Bland-Altman plots, solid line represents mean difference and dashed lines (B and D) are ±1.96SD

In order to eliminate the bias seen with the published model, a local linear regression equation was determined from the blood pool native T1 and the measured Hct. The following locally derived linear regression model was created: [HctMOLLI = (315.1 · [1/T1_blood_]) – 0.213]. (Fig. 2). While the calculated local model synthetic Hct had a similar fit with the measured Hct (r^2^ = 0.16, *p* < 0.001, Fig. 3a), the mean synthetic Hct was equivalent to the measured Hct at 41.8% ± 1.4% (*p* < 0.001, Table 2). The difference between measured and synthetic Hct ranged from −8.5% to 12.2%. Bland-Altman analysis of the local model demonstrated elimination of the bias seen in the published model, although there was a tendency for there to be greater discrepancy between measured Hct and synthetic Hct at the extremes (Fig. 3b). There was improved correlation by linear regression for both synthetic ECV at the mid-free wall (r^2^ = 0.87, *p* < 0.001, Fig. 2c) and the mid-septum (r^2^ = 0.83, *p* < 0.001, Additional file 2: Figure S2A). Minimal bias was observed on Bland-Altman analysis for either the mid-free wall ECV (Fig. 3d) or the mid-septum ECV (Additional file 2: Figure S2B). There was substantial variation in difference between measured and synthetic ECV, ranging from −8.4% to 4.3% for the mid-septum and from −12.6% to 15.8% for the mid-free wall.

### Static Hct estimation demonstrates strong ECV correlation

Previous studies have used the partition coefficient, which excludes the (1-Hct) term in the ECV equation, to characterize myocardial extracellular matrix expansion [26, 27]. For this model, ECV calculations were performed using a static Hct value of 45% for all ECV calculations, which is statistically equivalent to using the partition coefficient for linear regression analyses. Similar regression fit was seen compared to the other synthetic ECV models at the mid-free wall (r^2^ = 0.85, *p* < 0.001, Additional file 3: Figure S3A) and the mid-septum (r^2^ = 0.80, *p* < 0.001). Bland-Altman analysis demonstrated a modestly larger 1.6% bias (Additional file 3: Figure S3B) in the calculated synthetic ECV at the mid-free wall. There was substantial variation in the difference between measured and synthetic ECV, ranging from −8.0% to 6.0% for the mid-septum and from −8.0% to 6.3% for the mid-free wall.

### Modest intraclass correlation for Hct but excellent for ECV

ICCs were calculated for Hct and the ECV at mid-septum and mid-free wall using the published model, our local model, and the static Hct equal to 45% as an estimation of the partition coefficient (Table 3). The published model showed modest correlation for Hct overall as well as in control, muscular dystrophy, and congenital subpopulations. The ICCs for mid-septum and mid-free wall ECV with this model were substantially better. For the local model, the ICC for Hct was worse than the published model, however the ICCs for ECV for both mid-septum and mid-free wall were slightly better than the published model. Use of the partition coefficient demonstrated similar results with strong ICCs.

**Table 3:** Intraclass correlation coefficients (ICCs) for the three models

	Published Model		Static Hct (Partition Coefficient)		Local Model	
	*ICC*	*95% CI*	*ICC*	*95% CI*	*ICC*	*95% CI*
Hct_all_ (*n* = 163)	0.40	[0.27, 0.52]	---	---	0.28	[0.13, 0.42]
Hct_same day_ (*n* = 146)	0.43	[0.29, 0.56]	---	---	0.32	[0.17, 0.46]
Hct_control_ (*n* = 18)	0.54	[0.12, 0.80]	---	---	0.25	[−0.23, 0.63]
Hct_DMD_ (*n* = 86)	0.38	[0.18. 0.54]	---	---	0.27	[0.07, 0.46]
Hct_congenital_ (*n* = 24)	0.33	[−0.07, 0.64]	---	---	0.31	[−0.10, 0.63]
ECV_all mid-septum_ (*n* = 159)	0.85	[0.80, 0.89]	0.89	[0.85, 0.92]	0.91	[0.88, 0.93]
ECV_same day mid-septum_ (*n* = 142)	0.83	[0.77, 0.88]	0.88	[0.84, 0.91]	0.90	[0.87, 0.93]
ECV_control mid-septum_ (*n* = 18)	0.94	[0.85, 0.98]	0.88	[0.69, 0.95]	0.92	[0.79, 0.97]
ECV_DMD mid-septum_ (*n* = 86)	0.86	[0.81, 0.91]	0.92	[0.87, 0.95]	0.93	[0.89, 0.95]
ECV_congenital mid-septum_ (*n* = 24)	0.82	[0.63, 0.92]	0.86	[0.71, 0.94]	0.89	[0.76, 0.95]
ECV_all mid-free wall_ (*n* = 159)	0.93	[0.91, 0.95]	0.92	[0.89, 0.94]	0.93	[0.91, 0.95]
ECV_same day mid-free wall_ (*n* = 142)	0.90	[0.87, 0.93]	0.92	[0.89, 0.94]	0.94	[0.92, 0.96]
ECV_control mid-free wall_ (*n* = 18)	0.97	[0.93, 0.99]	0.94	[0.84, 0.98]	0.96	[0.90, 0.99]
ECV_DMD mid-free wall_ (*n* = 86)	0.89	[0.84, 0.93]	0.94	[0.90, 0.96]	0.94	[0.92, 0.96]
ECV_congenital mid-free wall_ (*n* = 24)	0.83	[0.64, 0.92]	0.85	[0.68, 0.93]	0.88	[0.73, 0.94]

ICCs for Hct in the entire cohort (Hct_all_) as well as subsets of controls, DMD patients, and patients with congenital heart disease were all higher for the published model compared to the local model, though the confidence intervals suggest the differences may not be significant. There was no significant difference in ICCs between these subgroups for either model. The ICCs for ECV_mid-septum_ and ECV_mid-free wall_ for all three models were comparably strong, with the local model marginally better for the overall cohort. ICCs were not significantly different when calculated with or without same day Hct sub-group included

### Use of synthetic ECV may lead to clinically significant errors

Using our lab’s cutoff for abnormal ECV of 28.5%, we evaluated each of the models for accuracy compared to the measured ECV (Tables 4 and Additional file 5: Table S4). All three of the models demonstrated significant miscategorization of patients, with total fraction miscategorized ranging from 37% for the published model to 23% for the local model. While the local model had fewer errors in categorizing patients (27 and 37% fewer errors compared to the partition coefficient and published models, respectively), a larger proportion of these were false positives and the absolute number of clinically significant miscategorizations was still high. The rates of miscategorization were similar for patients with or without same day Hct (Tables 4 and Additional file 4: Table S4). Repeat analysis using 27.0% (2 standard deviations above the mean) as an abnormal ECV cut-off demonstrated a similar distribution of miscategorizations (Table 4). There were slightly fewer unique miscategorizations due to the fact that some of the borderline abnormal cases shifted solidly into the abnormal category with the decreased threshold.

**Table 4:** Clinical miscategorization of abnormal ECV in the three models

	Published Model		Static ECV		Local Model	
	ECV_mid-septum_	ECV_mid-free wall_	ECV_mid-septum_	ECV_mid-free wall_	ECV_mid-septum_	ECV_mid-free wall_
False Negative	41 (30)	26 (19)	34 (32)	26 (24)	14 (9)	12 (6)
False Positive	4 (8)	3 (2)	3 (3)	1 (2)	12 (11)	8 (6)
Both false negative	14 (9)		12 (12)		6 (2)	
Both false positive	1 (0)		1 (0)		3 (3)	
Total unique miscategorizations	59 (50)		51 (49)		37 (27)	

Using a threshold ECV of 28.5% for abnormal (3 SD), the number of false negatives and false positives were determined for each model

The local model had substantially fewer total miscategorizations, although at the expense of an increased frequency of false positives. Numbers in parentheses are the number of instances in each classification using a threshold of 27.0% (2 SD)

## Discussion

A growing body of evidence indicates that ECV is a useful non-invasive biomarker for diffuse extracellular matrix expansion, which is a significant pathological finding in many forms of myocardial diseases [7–10, 18, 28]. However, ECV is limited by the need for blood sampling in order to obtain an Hct for the calculation and the need for off-line processing. This has led to attempts to eliminate the need for measured Hct through estimation of a synthetic Hct and some sequences are using this synthetic Hct in-line to calculate an ECV based on the observed linear relationship between Hct and R1. However, the clinical accuracy of this method has yet to be established, particularly in pediatric patient populations.

Our data suggest that there may be a number of potential clinically relevant considerations when using synthetic ECV. One consideration is the reliability of synthetic Hct estimations. The ICC for synthetic Hct calculated with both the previously published regression equation and the locally derived equation were modest at best. Some of this variability may be due to timing of blood sampling, as recent studies have demonstrated significant Hct variation between measures only a few hours apart and that values may even be altered by position [29, 30]. However, while repeat analysis excluding patients without a same day Hct showed a slight improvement in fit for Hct (r^2^ = 0.19, *p* < 0.001, Additional file 5: Figure S5), ECV_Free wall_ (r^2^ = 0.88, *p* < 0.001, Additional file 6: Figure S6), and ICCs, there was no significant difference in the ECV correlations (Table 3). At first blush, it appears counterintuitive that the agreement for ECV is much better than for Hct. However, this is because there are four additional terms in the ECV calculation that are remaining constant. Thus, relatively large changes in Hct lead to smaller changes in the ECV as the effect is mitigated by the other variables in the equation.

Other potential confounding factors considered include age, BSA, muscular dystrophy, and presence of structural heart disease (Table S7). None of these factors significantly influence ICC or regression fit. While a number of factors could theoretically contribute to the bias noted in the published model fit of our data, we postulate that this is likely due to variations in individual magnets. In fact, a recent report in the adult literature showed a similar bias [30]. Generally speaking, the ICCs for Hcts and ECVs are within the confidence intervals across models and therefore are not significantly different. There is a bias introduced using the published model that is eliminated with the local model. However, while the local model corrects that bias, the local model is “less accurate” at extremes of Hct. We suspect that the inaccuracy at extremes is decreasing the ICC for Hct of the local model, while the correction of the bias is the reason that the local model has a higher ICC for the ECV results and a lower rate of miscategorization. Thus, it would be prudent for centers calculating synthetic Hct and ECV to determine this equation for each magnet in order to help eliminate possible bias.

Another more substantial concern is the significant variation seen between measured and synthetic ECVs for individual patients in both the local and published models. Up to 37% of patients had at least one false negative or false positive in one or both ECVs (mid-septum and mid-free wall) despite excellent linear regression fit. This considerably limits the clinical utility of using synthetic Hct and ECV estimation and could lead to delay in treatment for some and unnecessary worry and testing in others. As some CMR sequences now include in-line estimations of Hct in their software packages, caution should be taken in using these results in pediatric patients for clinical decision making if a measured Hct is not available.

As demonstrated by the strong ICCs for mid-septal and mid-free wall ECV in all models examined in this study, the differences in individual patients tend to washout over a larger cohort. Thus synthetic Hct and ECV may have some utility in large retrospective research cohorts where measured Hct data are not available. However, caution should be taken if looking at individual patient data in such research cohorts, and validation with measured Hct should be made when available.

## Limitations

While to our knowledge this is the largest study looking at synthetic ECV in pediatric populations, there are several limitations that should be considered. The cohort has a large number of muscular dystrophy patients as our institution has a large population of these patients who receive frequent CMRs. While there is certainly a risk of sampling bias, there was no difference in the ICC for muscular dystrophy patients with any of the models studied compared to controls or the cohort as a whole. Ninety percent of the patients had an Hct drawn on the day of their CMR and most but not all of these Hcts were drawn immediately prior to the CMR. Given that Hct has been shown to vary by up to 10% even when measured hours apart, this may be a possible source of error. As exclusion of patients without same day Hct values did not have a significant effect on correlation, this limitation is unlikely to have altered our conclusions.

## Conclusions

To our knowledge, this is the first study to investigate the accuracy and clinical applicability of synthetic Hct and ECV in a pediatric population. Our results suggest that ECV calculated from synthetic Hct may be a useful tool in large pediatric research cohorts, especially for retrospective analysis where a measured Hct value may not be available. In our population, however, the use of synthetic Hct for calculation of ECV results in clinically significant miscategorization of individual patients. Thus at this time, we recommend formal measurement of Hct in children and young adults for calculation of ECV in clinical CMRs and in small research cohorts. If synthetic ECV is to be used in clinical or research settings where measured Hct cannot be obtained, we recommend using a locally derived synthetic Hct regression model for the particular magnet being used for the CMR. Additionally, clinical decisions based on this method should be interpreted with caution.

## Additional files

Additional file 1: Fig. S1. Linear regression fit of measured vs synthetic ECV at mid-septum for published model. Similar regression fit to that of synthetic ECV at the mid-free wall (A) with similar 1.2% bias on Bland-Altman analysis (B). Dashed line in A represents line of identity. For Bland-Altman plot, solid line represents mean difference and dashed Dotted lines in Bland-Altman plot (B) are ±1.96SD. (PDF 227 kb)

Additional file 2: Fig. S2. Linear regression fit of measured vs synthetic ECV at mid-septum for local model. Excellent fit similar to that seen for ECV at the mid-free wall (A) with minimal bias on Bland-Altman analysis (B). Dashed line in A represents line of identity. For Bland-Altman plot, solid line represents mean difference and dashed lines (B) are ±1.96SD. (PDF 154 kb)

Additional file 3: Fig. S3. Linear regression fit of measured vs synthetic ECV at mid-free wall for static hematocrit = 45% model (partition coefficient). Similar regression fit to that of other synthetic ECV models at the mid-free wall (A) with slightly larger 1.6% bias on Bland-Altman analysis (B). Dashed line in A represents line of identity. For Bland-Altman plots, solid line represents mean difference and dashed lines (B) are ±1.96SD. (PDF 192 kb)

Additional file 4: Table S4. Clinical miscategorization of abnormal ECV in the three models for patients with same-day Hct. The number of false negatives and positives were determined by repeat analysis in only patients with same-day Hct values, again using a threshold ECV of 28.5% for abnormal (3 SD). The distribution of miscategoriations in patients with same day Hct was similar to that of the total population. The local model had substantially fewer total miscategorizations, although at the expense of an increased frequency of false positives. (DOCX 15 kb)

Additional file 5: Fig. S5. Linear regression fit of measured vs synthetic Hct for published and local models excluding CMRs without same day Hct. Slightly improved but still poor regression fits for Hct using the published model (A) and local model (C) when CMRs without same day Hct values available. Negative bias seen on Bland-Altman analysis of published model similar to that observed for the full cohort (B). Elimination of bias similar to that observed with full cohort seen on Bland-Altman analysis of local model. Dashed line in A and C represents line of identity. For Bland-Altman plots, solid line represents mean difference and dashed lines (B and D) are ±1.96SD. (PDF 206 kb)

Additional file 6: Fig. S6. Linear regression fit of measured vs synthetic ECVFree Wall for published and local models excluding CMRs without same day Hct. Slightly improved but still excellent regression fits for ECVFree Wall using the published model (A) and local model (C) when CMRs without same day Hct values available. Posiotive bias seen on Bland-Altman analysis of published model similar to that observed for the full cohort (B). Elimination of bias similar to that observed with full cohort seen on Bland-Altman analysis of local model. Dashed line in A and C represents line of identity. For Bland-Altman plots, solid line represents mean difference and dashed lines (B and D) are ±1.96SD. (PDF 332 kb)

## Abbreviations

bSSFP: Balanced steady state free precession
CMR: Cardiovascular magnetic resonance
DMD: Duchenne muscular dystrophy
ECM: Extracellular matrix
ECV: Extracellular volume fraction
Hct: Hematocrit
ICC: Intraclass correlation coefficient
LGE: Late gadolinium enhancement
LV: Left ventricle
MOLLI: MOdified Look-Locker Inversion
R1: Longitudinal relaxivity
T1: Longitudinal relaxation time constant
