# Supplementary material for: Synthetic hematocrit derived from the longitudinal relaxation of blood can lead to clinically significant errors in measurement of extracellular volume fraction in pediatric and young adult patients
Source: J Cardiovasc Magn Reson. 2017 Aug 2;19:58. doi: 10.1186/s12968-017-0377-z (PMC5541652; doi:10.1186/s12968-017-0377-z)
Supplement: Supplementary file 1 — Linear regression fit of measured vs synthetic ECV at mid-septum for published model. Similar regression fit to that of synthetic ECV at the mid-free wall (A) with similar 1.2% bias on Bland-Altman analysis (B). Dashed line in A represents line of identity. For Bland-Altman plot, solid line represents mean difference and dashed Dotted lines in Bland-Altman plot (B) are ±1.96SD. (PDF 227 kb) [file 12968_2017_377_MOESM1_ESM.pdf]

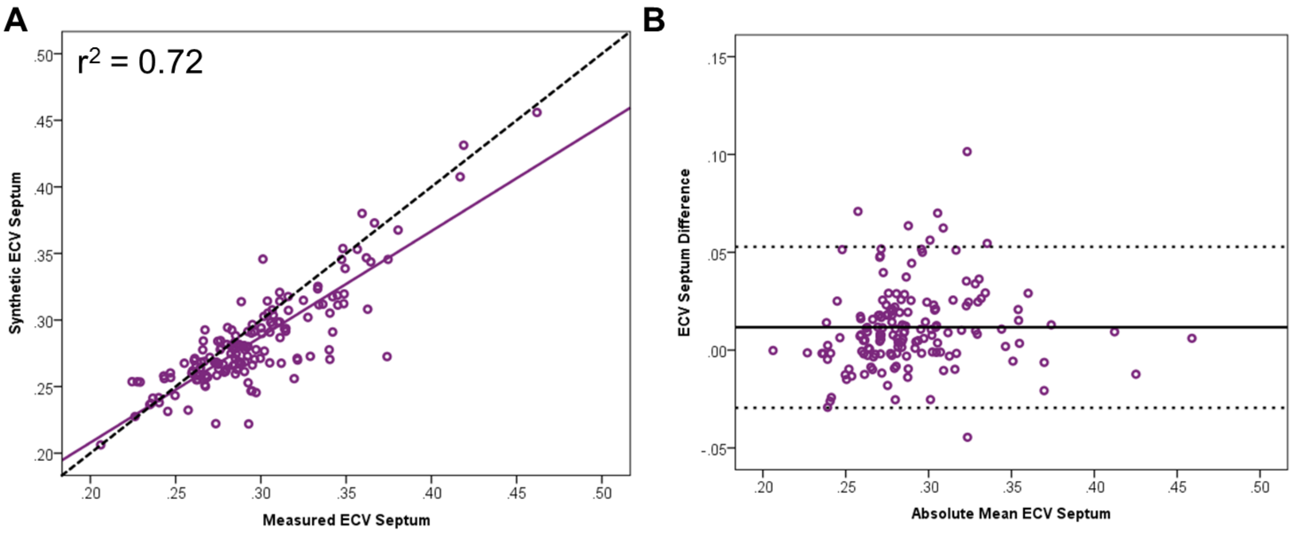


**Figure S1: Linear regression fit of measured vs synthetic ECV at mid-septum for published model.** Similar regression fit to that of synthetic ECV at the mid-free wall (A) with similar 1.2% bias on Bland-Altman analysis (B). Dashed line in A represents line of identity. For Bland-Altman plot, solid line represents mean difference and dashed Dotted lines in Bland-Altman plot (B) are ± 1.96SD.
