# Supplementary material for: Synthetic hematocrit derived from the longitudinal relaxation of blood can lead to clinically significant errors in measurement of extracellular volume fraction in pediatric and young adult patients
Source: J Cardiovasc Magn Reson. 2017 Aug 2;19:58. doi: 10.1186/s12968-017-0377-z (PMC5541652; doi:10.1186/s12968-017-0377-z)
Supplement: Supplementary file 2 — Linear regression fit of measured vs synthetic ECV at mid-septum for local model. Excellent fit similar to that seen for ECV at the mid-free wall (A) with minimal bias on Bland-Altman analysis (B). Dashed line in A represents line of identity. For Bland-Altman plot, solid line represents mean difference and dashed lines (B) are ±1.96SD. (PDF 154 kb) [file 12968_2017_377_MOESM2_ESM.pdf]

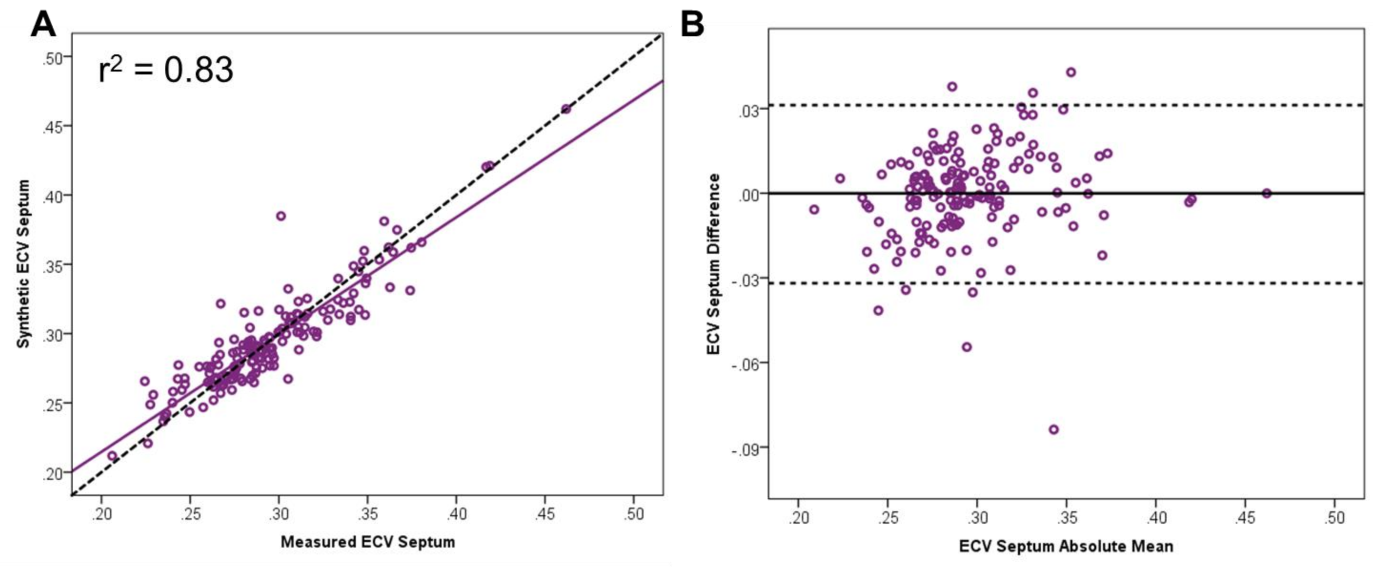

Figure S2: Linear regression fit of measured vs synthetic ECV at mid-septum for local model. Excellent fit similar to that seen for ECV at the mid-free wall (A) with minimal bias on Bland-Altman analysis (B). Dashed line in A represents line of identity. For Bland-Altman plot, solid line represents mean difference and dashed lines (B) are ± 1.96SD.
