# Supplementary material for: Synthetic hematocrit derived from the longitudinal relaxation of blood can lead to clinically significant errors in measurement of extracellular volume fraction in pediatric and young adult patients
Source: J Cardiovasc Magn Reson. 2017 Aug 2;19:58. doi: 10.1186/s12968-017-0377-z (PMC5541652; doi:10.1186/s12968-017-0377-z)
Supplement: Supplementary file 4 — Clinical miscategorization of abnormal ECV in the three models for patients with same-day Hct. The number of false negatives and positives were determined by repeat analysis in only patients with same-day Hct values, again using a threshold ECV of 28.5% for abnormal (3 SD). The distribution of miscategoriations in patients with same day Hct was similar to that of the total population. The local model had substantially fewer total miscategorizations, although at the expense of an increased frequency of false positives. (DOCX 15 kb) [file 12968_2017_377_MOESM4_ESM.docx]

**Table S4: Clinical miscategorization of abnormal ECV in the three models for patients with same-day Hct**. The number of false negatives and positives were determined by repeat analysis in only patients with same-day Hct values, again using a threshold ECV of 28.5% for abnormal (3 SD). The distribution of miscategoriations in patients with same day Hct was similar to that of the total population. The local model had substantially fewer total miscategorizations, although at the expense of an increased frequency of false positives.

|  | Published Model | | Static ECV | | Local Model | |
| --- | --- | --- | --- | --- | --- | --- |
|  | ECV_mid-septum_ | ECV_mid-free wall_ | ECV_mid-septum_ | ECV_mid-free wall_ | ECV_mid-septum_ | ECV_mid-free wall_ |
| False Negative | 37 | 24 | 31 | 24 | 11 | 10 |
| False Positive | 4 | 3 | 2 | 1 | 12 | 8 |
| Both false negative | 13 |  | 11 |  | 5 |  |
| Both false positive | 1 |  | 1 |  | 3 |  |
| Total unique miscategorizations | 54 |  | 46 |  | 33 |  |
