# Supplementary material for: Synthetic hematocrit derived from the longitudinal relaxation of blood can lead to clinically significant errors in measurement of extracellular volume fraction in pediatric and young adult patients
Source: J Cardiovasc Magn Reson. 2017 Aug 2;19:58. doi: 10.1186/s12968-017-0377-z (PMC5541652; doi:10.1186/s12968-017-0377-z)
Supplement: Supplementary file 5 — Linear regression fit of measured vs synthetic Hct for published and local models excluding CMRs without same day Hct. Slightly improved but still poor regression fits for Hct using the published model (A) and local model (C) when CMRs without same day Hct values available. Negative bias seen on Bland-Altman analysis of published model similar to that observed for the full cohort (B). Elimination of bias similar to that observed with full cohort seen on Bland-Altman analysis of local model. Dashed line in A and C represents line of identity. For Bland-Altman plots, solid line represents mean difference and dashed lines (B and D) are ±1.96SD. (PDF 206 kb) [file 12968_2017_377_MOESM5_ESM.pdf]

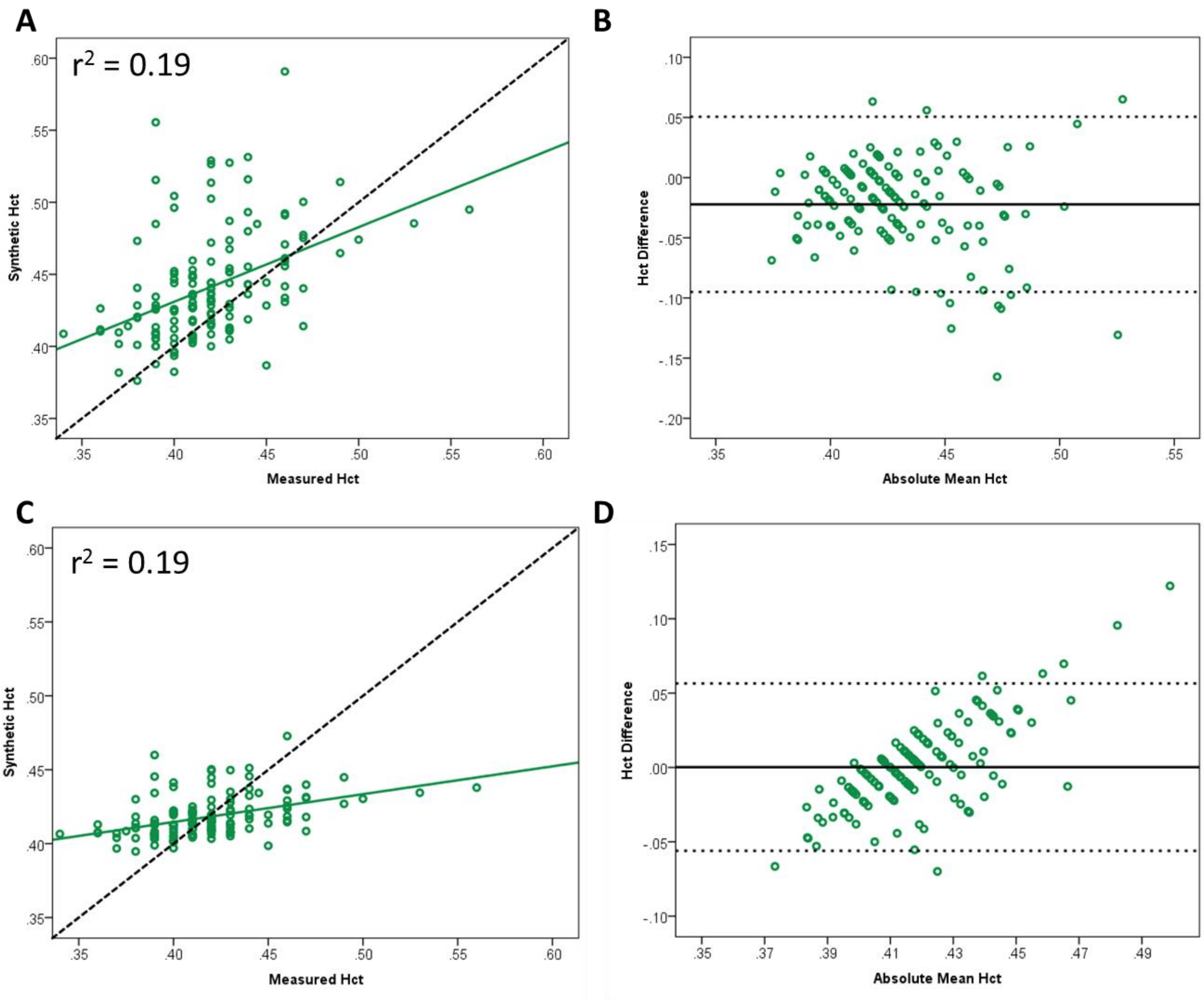


**Figure S5: Linear regression fit of measured vs synthetic Hct for published and local models excluding CMRs without same day Hct**. Slightly improved but still poor regression fits for Hct using the published model (A) and local model (C) when CMRs without same day Hct values available. Negative bias seen on Bland-Altman analysis of published model similar to that observed for the full cohort (B). Elimination of bias similar to that observed with full cohort seen on Bland-Altman analysis of local model. Dashed line in A and C represents line of identity. For Bland-Altman plots, solid line represents mean difference and dashed lines (B and D) are ± 1.96SD.
