# Supplementary material for: Synthetic hematocrit derived from the longitudinal relaxation of blood can lead to clinically significant errors in measurement of extracellular volume fraction in pediatric and young adult patients
Source: J Cardiovasc Magn Reson. 2017 Aug 2;19:58. doi: 10.1186/s12968-017-0377-z (PMC5541652; doi:10.1186/s12968-017-0377-z)
Supplement: Supplementary file 6 — Linear regression fit of measured vs synthetic ECVFree Wall for published and local models excluding CMRs without same day Hct. Slightly improved but still excellent regression fits for ECVFree Wall using the published model (A) and local model (C) when CMRs without same day Hct values available. Posiotive bias seen on Bland-Altman analysis of published model similar to that observed for the full cohort (B). Elimination of bias similar to that observed with full cohort seen on Bland-Altman analysis of local model. Dashed line in A and C represents line of identity. For Bland-Altman plots, solid line represents mean difference and dashed lines (B and D) are ±1.96SD. (PDF 332 kb) [file 12968_2017_377_MOESM6_ESM.pdf]

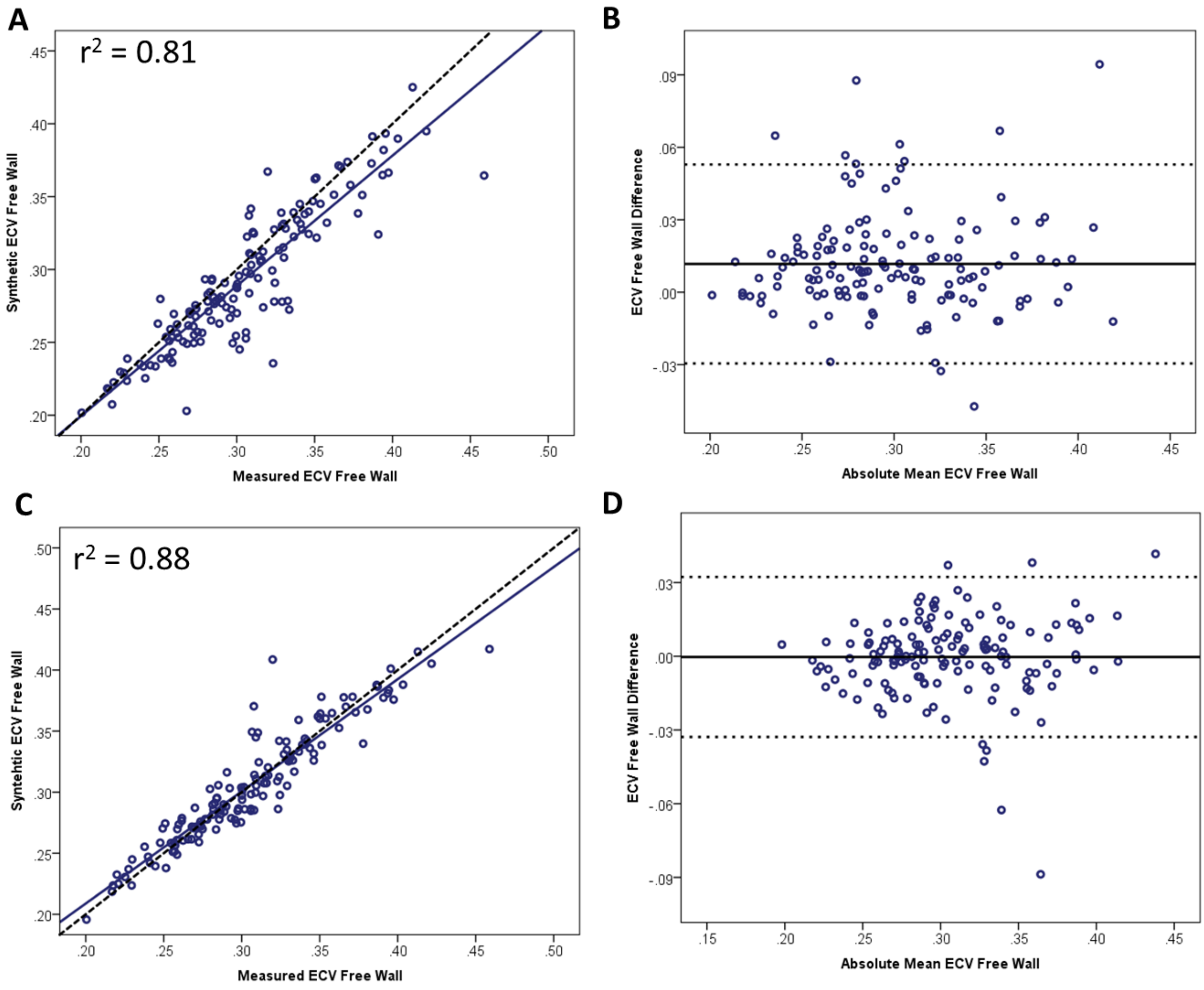

Figure S6: Linear regression fit of measured vs synthetic $ECV_{Free\ Wall}$ for published and local models excluding CMRs without same day Hct. Slightly improved but still excellent regression fits for $ECV_{Free\ Wall}$ using the published model (A) and local model (C) when CMRs without same day Hct values available. Posiotive bias seen on Bland-Altman analysis of published model similar to that observed for the full cohort (B). Elimination of bias similar to that observed with full cohort seen on Bland-Altman analysis of local model. Dashed line in A and C represents line of identity. For Bland-Altman plots, solid line represents mean difference and dashed lines (B and D) are ± 1.96SD.
